# Cognitive Symptoms in Cross-Sectional Parkinson Disease Cohort Evaluated by Human-in-the-Loop Machine Learning and Natural Language Processing

**DOI:** 10.1212/CPJ.0000000000200334

**Published:** 2024-07-02

**Authors:** Jennifer L. Purks, Lakshmi Arbatti, Abhishek Hosamath, Amy W. Amara, Karen E. Anderson, Lana Chahine, Shirley W. Eberly, Daniel Kinel, Sneha Mantri, Soania Mathur, David Oakes, David G. Standaert, Daniel Weintraub, Ira Shoulson, Connie Marras

**Affiliations:** Department of Neurology (JLP, DK, IS), University of Rochester, NY; Grey Matter Technologies (LA, AH, IS), a wholly owned subsidiary of Modality.ai, San Francisco, CA; Department of Neurology (AWA), University of Colorado Anschutz Medical Campus, Aurora; Departments of Psychiatry and Neurology (KEA), Georgetown University, Washington, DC; Department of Neurology (LC), University of Pittsburgh, PA; Department of Biostatistics and Computational Biology (SWE, DO), University of Rochester, NY; Department of Neurology (Sneha Mantri), Duke University, Durham, NC; PD Avengers (Soania Mathur), Toronto, Ontario, Canada; Department of Neurology (DGS), University of Alabama at Birmingham; Departments of Psychiatry and Neurology (DW), Perelman School of Medicine at the University of Pennsylvania, Philadelphia; and Edmond J Safra Program in Parkinson's Disease (CM), University Health Network, University of Toronto, Ontario, Canada.

## Abstract

**Background and Objectives:**

Cognitive impairment is experienced by up to 80% of people with Parkinson disease (PD). Little is known regarding the subjective experience and frequency of bothersome cognitive problems across the range of disease duration as expressed directly in patients' own words. We describe the types and frequency of bothersome cognitive symptoms reported verbatim by patients with PD.

**Methods:**

Through the online Fox Insight study and the Parkinson Disease Patient Report of Problems, we asked patients with PD to self-report by keyboard entry up to five most bothersome problems and how these problems affect their functioning. Human-in-the-loop curation, natural language processing, and machine learning were used to categorize responses into 8 cognitive symptoms: memory, concentration/attention, cognitive slowing, language/word finding, mental alertness/awareness, visuospatial abilities, executive abilities/working memory, and cognitive impairment not otherwise specified. Associations between cognitive symptoms and demographic and disease-related variables were examined in our cross-sectional cohort using multivariate logistic regression.

**Results:**

Among 25,192 participants (55% men) of median age 67 years and 3 years since diagnosis (YSD), 8,001 (32%) reported a cognitive symptom at baseline. The 3 most frequently reported symptoms were memory (13%), language/word finding (12%), and concentration/attention (9%). Depression was significantly associated with bothersome cognitive problems in all domains except visuospatial abilities. Predictors of reporting any cognitive symptom in PD were depression (adjusted OR 1.5), increasing MDS-UPDRS Part II score (OR 1.4 per 10-point increment), higher education (OR 1.2 per year), and YSD 1, 2, 6–7, and 8–9 vs 0 YSD. Among individuals with at least one cognitive symptom, posterior cortical-related cognitive symptoms (i.e., visuospatial, memory, and language) were reported by 17% (n = 4325), frontostriatal-related symptoms (i.e., executive abilities, concentration/attention) by 7% (n = 1,827), and both by 14.2% (n = 1,020). Odds of reporting posterior cortical symptoms vs frontostriatal symptoms increased with age and MDS-UPDRS part II score, but not depression.

**Discussion:**

Nearly one-third of participants with PD, even early in the disease course, report cognitive symptoms as among their most bothersome problems. Online verbatim reporting analyzed by human-in-the-loop curation, natural language processing, and machine learning is feasible on a large scale and allows a detailed examination of the nature and distribution of cognitive symptoms in PD.

## Introduction

Cognitive impairment in Parkinson disease (PD) is heterogeneous and associated with poor quality of life (QoL) for both people with PD and care partners.^1-4^ What is known about cognition in PD is largely derived from cognitive screening instruments or neuropsychological testing. The frequency of cognitive impairment as measured by neuropsychological tests and cognitive screening instruments increases with disease duration and severity,^5^ but differences in cognitive performance on neuropsychological tests as compared with non-PD controls can be detected even early in PD.^6^ Different cognitive profiles of cognitive impairment in PD have been defined.^6^ Yet, what patients with PD experience and report in their own words as cognitive impairment symptoms has been examined in very few studies involving small numbers of patients.^2,7-11^

We have developed machine learning algorithms based on natural language processing, machine learning, and human-in-the-loop curation to classify problems reported by patients in their own words through the PD-Patient Report of Problems (PD-PROP) and at an unprecedented scale.^12^ The combination of a large sample (>25,000 participants) and unconstrained free text reporting of problems allows us to observe a broader spectrum of bothersome problems in PD than was previously possible. In that work, we found that the domains represented by the ‘most bothersome’ problems in PD were (in descending order of frequency): other motor (most commonly impaired dexterity and speech), tremor, psychological, gait, balance, cognition, and pain (eFigure 1).^12^ We sought to 1) characterize the bothersome cognitive symptoms experienced by patients with PD and their frequencies and 2) describe associations of cognitive symptoms with demographic and disease characteristics in our large data set to better inform PD observational studies and patient-reported outcomes (PRO) as assessments for use in clinical trials. This report builds on previous PD-PROP analyses that focused on motor symptoms in this remarkably large cohort of research participants.^13,14^

## Methods

### Standard Protocol Approvals, Registrations, and Patient Consents

The Fox Insight study was approved by New England IRB. Informed consent was obtained through the Fox Insight website as part of the study by each patient before participation.

### Data Collection

The PD-PROP asks patients to report the most bothersome problems due to their PD and how these problems affect their functioning. As part of participation in Fox Insight,^15^ an online longitudinal cohort study involving over 25,000 participants, individuals with PD were asked to respond to the PD-PROP about every 3 months.^16^ Data used in the preparation of this article were obtained from the Fox Insight database^15^ on February 3, 2020.

### Data Curation

Curation is the process of identifying symptoms from problems expressed in patient verbatim response (“verbatims”) and classifying each verbatim as specifying or not specifying a given symptom. Symptom labels were determined by the curators, based on knowledge of PD symptoms. Details of the curation process can be found in the Supplementary Material. Based on review of approximately 3,500 verbatims, the curation process identified 8 symptoms: memory, concentration/attention, cognitive slowing, language/word finding, mental alertness/awareness, visuospatial abilities, executive abilities/working memory, and cognitive impairment not otherwise specified. Table 1 describes the conceptual boundaries of these symptoms. This experience provided terms and phrases that informed the development of an algorithm through a combination of natural language processing and machine learning, validated against the curator's classification as gold standard using a subset of verbatim responses. Accuracy (proportion of verbatims correctly classified) was 96% or higher for all cognitive domains. The entire process is depicted in eFigure 2 and described in detail in Marras et al.^12^

**Table 1:** Conceptual Boundaries for Cognitive Symptoms Identified in Curation

Domain	Proposed reported symptom	Conceptual boundaries	
		Includes	Excludes
Cognition	Memory	Impairment of memory including difficulty remembering information; learning new information; orientation to time, place	The term “having to remember”
	Concentration/attention	Difficulty concentrating or paying attention; sustaining focus	
	Cognitive slowing	Slowing or impairment of mental processing. Includes difficulty keeping up with conversations, slowness to respond, mental fatigue	Confusion, ‘brain fog’, mental sharpness
	Language/word finding	Difficulty understanding conversation; expressing oneself; difficulty speaking words that are being thought of. Difficulty understanding what is being read/reading	Difficulty understanding because of hearing impairment
	Mental alertness/awareness	Fluctuating alertness; fluctuations in/variable attention; zoning out, brain fog, confused thoughts, reduced mental sharpness	Cognitive/mental slowing
	Visuospatial abilities	Difficulty judging distances or depth; navigating 3-dimensional situations; orienting oneself in space; identifying visual and spatial relationships among objects; trouble navigating closed or indoor spaces that are familiar	Freezing (interruption of gait) in doorways or thresholds
	Executive abilities/working memory	Difficulty planning or executing tasks; multitasking; switching from one cognitive task to another, trouble following directions or instructions; problem solving; decision making; sequencing; learning new skills	
	Cognitive impairment NOS	Cognitive complaint not clearly fitting into another category. Could include confusion, muddled, mixed up	

### Cognitive Baseline Data and Statistical Analysis

Individuals with at least one PD-PROP report were included in this analysis. For participants with more than one PD-PROP, only the first report was used. Age, sex, years since diagnosis (YSD), Geriatric Depression Scale-15 (GDS-15) and Movement Disorder Society Unified Parkinson Disease Rating Scale Part II (MDS-UPDRS Part II) scores were obtained from Fox Insight. MDS-UPDRS Part II measures motor experiences of daily living.^17,18^ Scores range from 0-52, with increasing score representing greater disability. The maximum interval between the PD-PROP and MDS-UPDRS was 90 days. Depression was defined by GDS-15 score of 5 or greater.

Symptom frequencies (in %) were tabulated for each of the 8 cognitive symptoms by categories of age, sex, YSD, education, GDS-15, and MDS-UPDRS Part II. Differences across categories were tested using chi-square tests or Cochran-Armitage trend tests. *p* Values less than 0.05 were considered statistically significant. A logistic regression model including age, sex, YSD, education, GDS-15, and MDS-UPDRS Part II was created, with reporting of any cognitive symptom as the dependent variable.

### Frontostriatal and Posterior Cortical Analysis

Based on prior research suggesting 2 major neuroanatomical substrates of cognitive impairment in PD (frontostriatal and posterior cortical pathways)^19-22^ we explored if participants with symptoms attributable to frontostriatal vs posterior cortical dysfunction differed in demographic and PD clinical features. Executive abilities and concentration/attention symptom reports were grouped within the frontostriatal category, and visuospatial abilities, memory, and language/word finding symptom reports were grouped within the posterior-cortical category. Logistic regression was used to test associations between age, sex, YSD, education, depression, and MDS-UPDRS Part II and posterior cortical pathway associated symptoms, using frontostriatal associated symptoms as the reference group. Participants reporting symptoms in both categories or neither were reported as raw frequencies, but not specifically compared in the exploratory pathway odds ratio analysis.

### Data Availability

The data supporting the findings of the study are not publicly available because of privacy restrictions. The curated data sets derived from the verbatim responses are publicly available in FoxDEN^23^ upon signing a data use agreement.

## Results

A total of 25,192 Fox Insight participants who provided at least one PD-PROP report were included. Two participants with age >100 were excluded from the analysis. The characteristics of the participants are shown in Table 2. Eight thousand and one (32%) participants reported at least one cognitive symptom as a most bothersome problem. The 3 most frequently reported cognitive symptoms were memory (13%), language/word finding (12%), and concentration/attention (9%).

**Table 2:** Baseline Characteristics of Sample (N = 25,192)

Age	
< 50	1,520 (6%)
50–59	4,356 (17%)
60–69	9,608 (38%)
70–79	8,150 (32%)
≥ 80	1,558 (6%)
Sex	
Female	11,215 (45%)
Male	13,977 (55%)
Race	
White	23,534 (93%)
Black/African American	176 (1%)
American Indian/Alaskan Native	74 (<1%)
Asian	380 (2%)
Pacific Islander	13 (<1%)
Other/mixed/unknown	1,114 (4%)
Ethnicity	
Hispanic	1,047 (4%)
Not Hispanic	22,722 (90%)
Unknown	1,423 (6%)
Years since diagnosis	
0	3,503 (14%)
1	3,800 (15%)
2	3,114 (12%)
3–5	6,168 (25%)
6–7	2,562 (10%)
8–9	1,766 (7%)
≥ 10	4,222 (17%)
Education	
High School	2,779 (11%)
Associates/College	13,618 (56%)
Postgraduate	8,047 (33%)
Depression	
GDS-15 < 5	13,506 (63%)
GDS-15 ≥ 5	7,981 (37%)
MDS-UPDRS II (Quartiles)	
Q1: 0–6	6,338 (28%)
Q2: 7–11	5,769 (25%)
Q3: 12–17	5,404 (24%)
Q4: ≥ 18	5,180 (23%)
Cognitive slowing	
Not reported	24,085 (96%)
Reported	1,107 (4%)
Executive abilities/working memory	
Not reported	24,364 (97%)
Reported	828 (3%)
Concentration/attention	
Not reported	22,808 (91%)
Reported	2,384 (9%)
Memory	
Not reported	21,867 (87%)
Reported	3,325 (13%)
Language/word finding	
Not reported	22,060 (88%)
Reported	3,132 (12%)
Mental alertness/awareness	
Not reported	24,460 (97%)
Reported	732 (3%)
Cognitive impairment NOS	
Not reported	24,890 (99%)
Reported	302 (1%)
Visuospatial abilities	
Not reported	25,151 (>99%)
Reported	41 (<0.5%)
Any cognitive symptom	
Not reported	17,191 (68%)
Reported	8,001 (32%)

Abbreviation: NOS = not otherwise specified.

Values shown are N (%).

The unadjusted frequencies of each cognitive symptom by age, sex, YSD, education, and MDS-UPDRS Part II are shown in Table 3. Across age groups, the proportion of participants reporting at least one cognitive symptom (“any symptom”) was not significantly different. However, the distribution of individual cognitive symptoms varied across age groups. Concentration problems were reported less than half as frequently by individuals older than 80 years compared with those younger than 50 years while language symptoms were almost twice as commonly reported in individuals older than 80 years compared with those younger than 50 years. There were other statistically significant trends but the magnitude of frequency differences across the age range were smaller. Reporting frequency of all 8 cognitive symptoms increased with higher MDS-UPDRS II quartiles; memory and language showed the largest increases. No important differences in frequency by sex, educational attainment, or YSD were noted.

**Table 3:** Symptom Frequencies (% Reporting Problem) by Baseline Characteristics

	N	Cognitive slowing	Executive abilities	Concentration	Memory	Language	Mental alertness	Cognitive impairment NOS	Visuospatial	Any cognitive symptom
Age										
< 50	1,520	**5.6**	**4.2**	**13.5**	**12.7**	**9.5**	2.9	**0.8**	0.1	32.1
50–59	4,356	**4.9**	**4.0**	**11.8**	**11.6**	**10.5**	2.8	**1.0**	0.1	31.8
60–69	9,608	**4.7**	**3.4**	**9.6**	**12.9**	**12.9**	2.9	**1.1**	0.2	31.6
70–79	8,150	**3.6**	**2.8**	**7.9**	**13.9**	**13.0**	2.9	**1.4**	0.2	31.5
≥ 80	1,558	**4.0**	**2.1**	**6.4**	**16.3**	**15.1**	3.3	**1.9**	0.1	33.7
Sex										
Female	11,215	**3.8**	3.3	9.2	13.1	12.5	**3.2**	1.2	0.1	31.2
Male	13,977	**4.8**	3.3	9.7	13.2	12.4	**2.7**	1.2	0.2	32.2
Years since diagnosis										
0	3,503	**3.3**	**2.6**	**9.4**	12.9	**9.9**	3.1	1.1	0.0	**28.8**
1	3,800	**4.3**	**2.9**	**10.3**	14.6	**11.8**	2.9	1.3	0.1	**31.9**
2	3,114	**4.5**	**3.0**	**10.1**	13.4	**12.0**	2.7	1.0	0.1	**31.9**
3–5	6,168	**4.3**	**3.1**	**9.6**	12.9	**12.4**	2.7	1.3	0.2	**31.5**
6–7	2,562	**4.8**	**4.0**	**9.7**	12.8	**12.6**	3.2	0.8	0.3	**32.9**
8–9	1,766	**5.4**	**4.1**	**9.2**	13.4	**15.6**	3.1	1.2	0.1	**34.8**
≥ 10	4,222	**4.9**	**4.0**	**8.2**	12.7	**14.0**	3.0	1.4	0.2	**32.7**
Education										
High School	2,779	**3.0**	**2.3**	8.4	**14.0**	12.2	2.2	1.2	0.1	30.4
Assoc./College	13,618	**4.2**	**2.9**	9.6	**13.6**	12.9	3.1	1.3	0.2	32.2
Postgraduate	8,047	**5.1**	**4.1**	9.5	**12.4**	11.9	3.0	1.1	0.1	31.6
MDS-UPDRS II (Quartiles)										
Q1: 0–6	6,338	**2.6**	**2.4**	**8.0**	**9.7**	**8.2**	**2.3**	**0.5**	**0.1**	**23.3**
Q2: 7–11	5,769	**4.2**	**3.5**	**10.4**	**13.2**	**11.5**	**2.7**	**1.0**	**0.1**	**31.6**
Q3: 12–17	5,404	**5.2**	**4.3**	**10.8**	**14.5**	**14.2**	**3.5**	**1.4**	**0.2**	**35.6**
Q4: ≥ 18	5,180	**5.9**	**3.4**	**9.4**	**16.4**	**17.8**	**3.5**	**2.1**	**0.4**	**39.4**
Depression										
GDS-15 < 5	13,506	**3.5**	**2.9**	**8.3**	**10.3**	**10.9**	**2.6**	**0.8**	0.2	**27.3**
GDS-15 ≥ 5	7,981	**6.2**	**4.2**	**11.7**	**18.0**	**15.4**	**3.6**	**1.8**	0.2	**39.5**

Data shown are % reported. Bold indicates a significant difference across categories (*p* < 0.05, χ^2^ test or Cochran-Armitage trend test).

**Figure 1:**
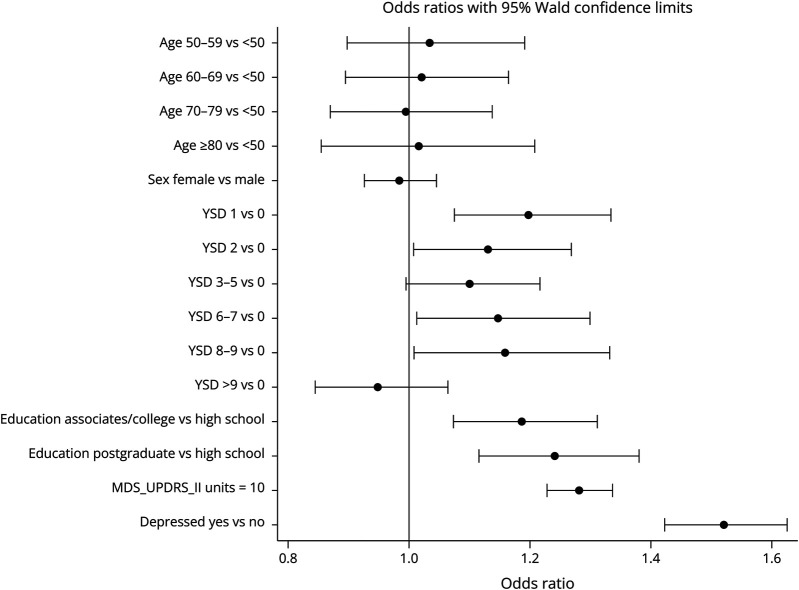
Adjusted OR of Any Cognitive Symptom Reported Compared With No Cognitive Symptom (Reference Group) Odds ratios (OR) shown are for an increment of 10 for MDS-UPDRS II. Variables of analysis include age, sex, years since diagnosis, education, MDS-UPDRS part II score, and depression (as measured by GDS-15).

The multivariable associations between age, sex, YSD, education, depression, MDS-UPDRS II and reporting any cognitive symptom are shown in Figure 1. In adjusted multivariable logistic regression analysis, depression was associated with the greatest odds of reporting a cognitive symptom (odds ratio 1.5) (Figure 1). In addition, greater MDS-UPDRS Part II, higher education, and a trend for YSD were associated with a greater odds of reporting a cognitive symptom as a most bothersome problem.

**Figure 2:**
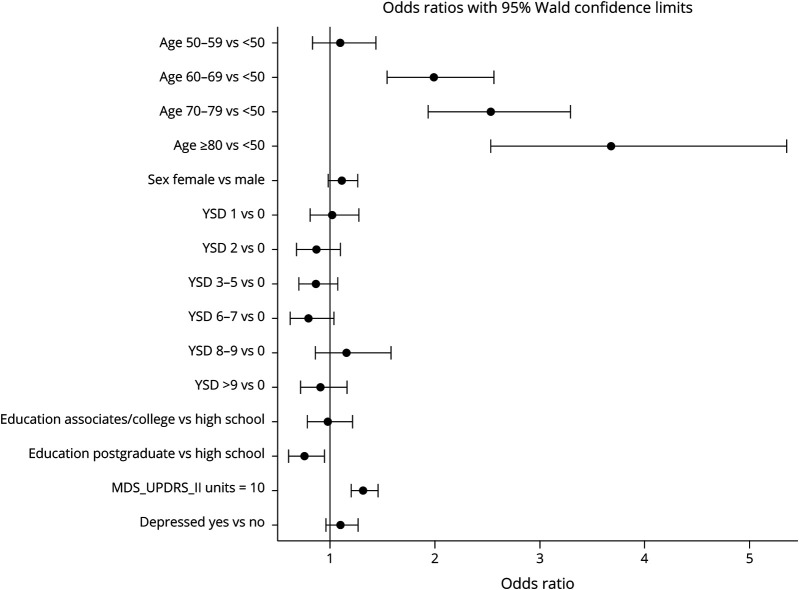
Adjusted OR of Posterior Cortical Deficits Compared With Frontostriatal Deficits (Reference Group) Odds ratios (OR) shown are for an increment of 10 for MDS-UPDRS II. Variables of analysis include age, sex, years since diagnosis, education, MDS-UPDRS part II score, and depression (as measured by GDS-15).

When PD-PROP cognitive responses were grouped by pathway, posterior cortical related symptoms (visuospatial, memory, and language) were reported by 17% (n = 4,325), frontostriatal related symptoms (executive abilities, concentration) by 7% (n = 1,827), both by 14.2% (n = 1,020), and neither (including cognitive slowing, mental alertness/awareness and cognitive impairment not otherwise specified symptoms) by 72% (18,020). The odds of posterior cortical complaints compared with frontostriatal complaints increased with age and MDS-UPDRS part II score (Figure 2).

## Discussion

Cognitive problems as a most bothersome symptom in PD were reported by one-third of our cohort, even in early disease. Our large cross sectional PD cohort findings add to a robust literature indicating that subjective cognitive complaints are frequent in PD.^7-11^ A systematic review and meta-analysis that included 3441 patients of varying disease duration and severity identified a very similar frequency of cognitive complaints (36%).^24^ We found that patients reported a broad array of cognitive concerns. Our study establishes cognitive problems as frequently among the most bothersome problems across the range of disease duration in PD.

To date, self-reported cognitive problems, often termed cognitive complaints, have been measured using screening questionnaires such as the PD Non-motor Symptom Questionnaire (NMSQuest),^25^ modified Neurobehavioral Inventory,^26^ the Non-Motor Symptom Scale (NMSS),^27^ or custom screening questions such as, “Do you feel that your memory and thinking have gotten worse?”^28^ Often, these measures solicit cognitive problems in a restricted number of domains^25^ or only memory.^28^ A recent systematic review and meta-analysis found wide ranges of frequency of subjective cognitive complaints based on how the question was asked. For example, when asked with a global question “Do you feel that you have a declining memory?” subjective cognitive complaint frequency can be up to 50% higher compared with questionnaires with several specific examples such as “Do you often forget appointments?”^24^ To our knowledge, our study examining the frequency and nature of subjective cognitive problems without prompting individuals about specific cognitive domains is unique.

Subjective cognitive complaints (SCCs) may alert clinicians to test for and identify actual cognitive impairment. Although not always concordant, subjective cognitive complaints are associated with objective cognitive impairment. A systematic review and meta-analysis of subjective cognitive complaints in PD found that SCCs were associated with poorer performance in global cognition, working memory and attention, executive function, language, memory, and visual spatial impairment.^24^ Although we had no measure of cognitive impairment in our study, it is interesting to put our results into the context of the prevalence of objective cognitive deficits in PD, recognizing that subjective cognitive concerns do not necessarily align with objective findings or predict worse cognitive outcomes.^26,29,30^ Across all years of PD duration, the rate of reporting cognitive issues as a most bothersome problem in our study was similar to the prevalence of objective cognitive deficit of 25%–40% reported in the literature.^2,4^ In the first few years after a PD diagnosis varying rates of objective cognitive impairment have been reported. In one study that included 53 newly diagnosed patients (defined as disease duration ≤1 year), 20 (37.7%) were classified as PD-MCI at baseline.^31^ In another study assessing objective visuospatial, executive, and memory function, 24–62% had deficits at diagnosis as compared with healthy controls at diagnosis.^20^

### Individual Cognitive Symptoms

Memory was the most frequently reported bothersome cognitive symptom (13%), followed closely by Language/word finding (12%) and Concentration/attention (9%). In qualitative interviews with people with PD, subjective symptoms queried in “off” periods included concerns related to concentration, “thinking,” and language, which partially align with our top 3 cognitive domains reported.^8^ Other literature suggests that memory concerns are a large contributor to symptom burden among patients with PD.^3^ The predominance of “memory” concerns could be related to the lay lexicon equating “memory” with cognitive problems. Similarly, the low frequency of other cognitive concerns may be influenced by a lack of familiarity with terminology to describe these aspects of cognitive function. These uncertainties notwithstanding, there are several reasons that may underlie subjective memory complaints, including true memory dysfunction, executive dysfunction manifesting as working memory impairment, anxiety, or depression. Cognitive and psychiatric screening and more in-depth cognitive testing as appropriate would be important to direct care for these patients.

### Patterns of Association

The relationship between cognitive symptoms and age and disease duration varied across domains while increasing motor dysfunction (MDS-UPDRS II used as a proxy) paralleled increasing cognitive problem reporting across all 8 cognitive domains. Concentration problems decreased with age, but memory and language complaints increased. This may reflect copathology (e.g., Alzheimer disease or vascular disease) and different demands (i.e., not working outside the home) influencing the experience of cognitive impairment. One of our key hypotheses based on current literature and clinical practice was that cognitive complaints would increase with YSD.^20^ However, this was not seen in this sample. Individuals within the first year since PD diagnosis had the highest odds of reporting a cognitive symptom as one of their most bothersome problems after adjusting for depression, age, sex, YSD, MDS-UPDRS Part II, and education, suggesting that patients with PD are most likely to worry about or prioritize cognitive concerns early in the disease course, although the differences in frequency across the YSD range were small. Furthermore, it is likely that some individuals early in their disease are reporting subjective cognitive concerns in the absence of objective cognitive decline. Noncognitive contributions to these reported experiences, such as depression or anxiety, should be considered.

It is also possible that the lack of association between cognitive problems and YSD reflects volunteer bias; the online and self-report nature of the study may exclude people with more significant cognitive problems seen in the later stages of disease. Other than the biology of PD, changing patient priorities, reduced insight, and even normalization of cognitive dysfunction may all influence the pattern of self-reported most bothersome problems as the disease progresses. Our study reports what patients perceive as their most bothersome problems which is influenced by every aspect of their PD progression. So as patients accumulate other cognitive and noncognitive problems, the priority of individual cognitive domain symptoms may change. For some symptoms, the frequency is slightly lower in the highest quintile of disease duration, which may be related to normalization or coping with progression process or aging. However, our cross-sectional analysis is not designed to capture if the reported symptom persists, only to assess its relative importance. There is little known from the prior literature regarding demographic and clinical correlates of SCC; however, a meta-analysis also found lower rates of SCC in more advanced disease and postulated changing priorities as an explanation.^24^

Higher education was positively associated with reporting of memory symptoms as a bothersome problem. Considering prior literature on objective cognitive performance, there are data to suggest that higher education may be protective against cognitive decline because of cognitive reserve.^31^ On the other hand, more years of formal education has also been associated with a state of “worried well" and a higher prevalence of cognitive concerns.^32^ More highly educated patients with PD may be more likely to report cognitive concerns because of greater insight into their symptoms or a greater observed loss in their day-to-day higher level function. Given that we do not have an objective measure of cognitive decline in this study, we are unable to establish which of these possibilities account for our findings.

The prior literature has extensively documented the association of PD cognitive symptoms with depression.^2,4,24^ This is consistent with our finding of an association between cognitive concerns and depression in all but the visuospatial domain. Of interest, there is previous suggestion of patients poorly articulating visuospatial concerns, which may account for this exception.^33^ Within individuals, if cognitive concerns are not accompanied by objective cognitive impairment, they may be indicators of noncognitive issues such as depression or anxiety.^4^ This is supported by a recent meta-analysis that demonstrated that all 3 have a moderate association with subjective cognitive complaints.^24^

### Frontostriatal and Posterior Cortical Patterns of Cognitive Problems

Existing literature suggests two neural pathways implicated in PD cognitive pathology. This classification distinguishes frontostriatal cognitive deficits (including executive abilities and concentration secondary to dopamine loss) from posterior cortical cognitive deficits (including visuospatial, memory, and language secondary to cholinergic loss).^19,20^ Prior studies have suggested a different pathophysiology, progression, and prognosis for frontostriatal compared with posterior cortical function.^21^ In a longitudinal 5-year analysis of a de novo PD cohort in the CamPaigIGN study, executive and frontostriatal function were not associated with development of dementia, but measures of posterior cortical function were correlated with development of PD dementia.^21^ Given that increasing age and posterior cortical deficits are associated with dementia,^5,20,21^ we hypothesized that younger patients may report more cognitive complaints in a frontostriatal pattern compared with posterior cortical deficit pattern and that older patients may report the opposite pattern. Indeed, we found that older individuals were more likely to voice posterior cortical rather than frontostriatal cognitive concerns, the likelihood increasing with each decade over 50 years. This matches the hypothesis that posterior cortical dysfunction may accompany age-related neurodegenerative cognitive disorders, such as concomitant Alzheimer disease pathology. Longitudinal examination of this cohort to assess the prognostic value of domain-specific cognitive concerns will be of value to guide cognitive monitoring and investigation.

### Predictive Value and Longitudinal Implications

Subjective, self-reported cognitive impairment has been found to capture earlier cognitive decline than standardized testing.^28^ Eliciting subjective cognitive concerns early may be useful to predict development of MCI,^2,22^ which is important given the predictive value of PD-MCI for the development of PD dementia.^34,35^ Identifying patients who are more likely to develop PD dementia is a key priority to facilitate treating at-risk patients once disease-modifying treatments are available. Furthermore, the literature provides robust evidence that cognitive deficits reduce QoL and function.^3,4,6^ Although subjective cognitive concerns do not necessarily align with objective findings or predict worse cognitive outcomes,^26,29,30^ longitudinal follow-up of these individuals has recently demonstrated that subjective cognitive concerns as elicited by the PD-PROP is associated with future development of cognitive functional impairment.^36^

### Limitations

This study benefits from a large unstructured patient-reported outcome data set, but there are some limitations. At times, patient reports were ambiguous, which in a clinical setting would be clarified with follow-up questions; because this was not possible because of the nature of data collection, misinterpretation or misclassification is possible. To mitigate this, curators used a cognitive impairment not otherwise specified category, but the actual nature of these concerns is unclear. Some of the cognitive categories are broad, and a more granular classification of reported problems would provide additional insights, such as distinguishing language comprehension and expression problems. Cognitive symptom classifications were largely anchored in predetermined symptoms based on the clinical and personal experiences of the curators. New symptoms were added if they were noted in the curation process; however, only ∼3,500 total verbatims were examined by the curators to guide these additions. In addition, without objective cognitive assessment to accompany patient reporting we do not know whether the subjective concerns accurately reflect cognitive deficits. Furthermore, the online nature of the study which relied on participant self-selection and self-motivation for ongoing participation leads to a potential volunteer bias. Particularly, individuals with longer disease duration and participating in our study will be a select group with more indolent disease, blunting the relationship between YSD and disease progression, both cognitive and motor. Furthermore, MDS-UPDRS II, which measures activities of daily living, was used as a proxy for motor disease severity. Finally, the nature of the data collection was not designed to directly interrogate cognitive pathways (posterior cortical vs frontostriatal); therefore, it is not possible to know whether most of the individuals fit into one or other of the hypothesized pathways. 72% of the sample could not be classified into either of the 2 proposed cognitive patterns; therefore, we may have missed other associations with these patterns that would be revealed by a different instrument.

### Conclusion

Large-scale free text responses analyzed with a combination of human-in-the-loop curation, natural language processing, and machine learning to develop an algorithm to classify symptoms is feasible to prioritize PD cognition symptoms. Our data set demonstrates a high and wide-ranging cognitive symptom burden for patients with PD that only partially quantifies this burden by asking only about most bothersome PD symptoms. The cognitive concerns begin early after diagnosis and have a parallel association with motor functional impairment and depression. The association of depression with reporting cognitive problems as a most bothersome problem for all domains except visual spatial symptoms demonstrates the importance of mood screening and treatment strategies targeted to the underlying source of the subjective cognitive concerns. Future directions include the need for both subjective cognitive concerns and objective cognitive assessment to be part of the same study.

**TAKE-HOME POINTS**

→ The combination of a large sample (>25,000 patients) and unconstrained free text reporting of problems allowed us to observe a broader spectrum of most bothersome cognitive problems in PD than was previously possible.
→ Free text responses analyzed with a combination of human-in-the-loop curation, natural language processing, and machine learning to develop an algorithm to classify symptoms is feasible to study the nature of cognitive change in PD as experienced by patients.
→ Nearly one-third of participants, including 14% (i.e., 3503) within the first year after PD diagnosis, reported cognitive symptoms as a most bothersome problem. Of any cognitive complaint reported, the 3 most frequent were memory (13%), language/word finding (12%), and concentration/attention (9%).
→ Depression (GDS-15 score of 5 or greater), higher MDS-UPDRS II (motor activities of daily living), and higher education, but not age were associated with reporting cognitive problems as a most bothersome problem.
→ The odds of posterior cortical complaints (i.e., visuospatial, memory, and language) compared with frontostriatal complaints (i.e., executive abilities, concentration/attention) increased with age and MDS-UPDRS part II score.

## Appendix Authors

**Table:**

Name	Location	Contribution
Jennifer L. Purks, MD	Department of Neurology, University of Rochester, NY	Drafting/revision of the manuscript for content, including medical writing for content; major role in study concept or design; analysis or interpretation of data
Lakshmi Arbatti, MS	Grey Matter Technologies, a wholly owned subsidiary of Modality.ai, San Francisco, CA	Revision of the manuscript for content, including writing the technical methodology for content; major role in the acquisition of data; study concept or design; analysis or interpretation of data
Abhishek Hosamath, MBM	Grey Matter Technologies, a wholly owned subsidiary of Modality.ai, San Francisco, CA	Revision of the manuscript for content, including writing the technical methodology for content; study concept or design; analysis or interpretation of data
Amy W. Amara, MD, PhD	Department of Neurology, University of Colorado Anschutz Medical Campus, Aurora	Revision of the manuscript for content, including medical writing for content; analysis or interpretation of data
Karen E. Anderson, MD	Departments of Psychiatry and Neurology, Georgetown University, Washington, DC	Revision of the manuscript for content, including medical writing for content
Lana Chahine, MD, MS	Department of Neurology, University of Pittsburgh, PA	Revision of the manuscript for content, including medical writing for content; study concept or design; analysis or interpretation of data
Shirley W. Eberly, BS, MS	Department of Biostatistics and Computational Biology, University of Rochester, NY	Revision of the manuscript for content, including medical writing for content; study concept or design; analysis or interpretation of data
Daniel Kinel, JD	Department of Neurology, University of Rochester, NY	Revision of the manuscript for content, including medical writing for content
Sneha Mantri, MD, MS	Department of Neurology, Duke University, Durham, NC	Revision of the manuscript for content, including medical writing for content; study concept or design; analysis or interpretation of data
Soania Mathur, MD	PD Avengers, Toronto, Ontario, Canada	Revision of the manuscript for content, including medical writing for content
David Oakes, PhD	Department of Biostatistics and Computational Biology, University of Rochester, NY	Revision of the manuscript for content, including medical writing for content; study concept or design; analysis or interpretation of data
David G. Standaert, MD, PhD	Department of Neurology, University of Alabama at Birmingham	Revision of the manuscript for content, including medical writing for content
Daniel Weintraub, MD	Departments of Psychiatry and Neurology, Perelman School of Medicine at the University of Pennsylvania, Philadelphia	Revision of the manuscript for content, including medical writing for content; analysis or interpretation of data
Ira Shoulson, MD	Department of Neurology, University of Rochester, NY; Grey Matter Technologies, a wholly owned subsidiary of Modality.ai, San Francisco, CA	Revision of the manuscript for content, including medical writing for content; major role in the acquisition of data; study concept or design; analysis or interpretation of data
Connie Marras, MD, PhD	Edmond J. Safra Program in Parkinson's disease, University Health Network, University of Toronto, Ontario, Canada	Drafting/revision of the manuscript for content, including medical writing for content; major role in study concept or design; analysis or interpretation of data
